# Optimization of Ultrasound‐Assisted Extraction Conditions for Phenolic Content and Antioxidant Activity in Green Mango Peel Using Response Surface Methodology

**DOI:** 10.1002/fsn3.71997

**Published:** 2026-06-09

**Authors:** Md. Arifur Rohman, Md. Naimur Rahman, Md. Abdul Halim, Mouluda Sohany, Md. Sultan Mahomud

**Affiliations:** ^1^ Department of Food Engineering and Technology Hajee Mohammad Danesh Science and Technology University Dinajpur Bangladesh; ^2^ Department of Nutrition and Food Engineering Daffodil International University, DSC, Birulia Savar Dhaka Bangladesh; ^3^ Department of Food Science and Nutrition Hajee Mohammad Danesh Science and Technology University Dinajpur Bangladesh

**Keywords:** antioxidant activity, bioactive compounds, mango peel, RSM

## Abstract

Green mango peel ( *Mangifera indica* L.) is an abundant agro‐industrial by‐product rich in phenolic compounds with strong antioxidant potential, making it a promising source of natural bioactives for food and nutraceutical applications. However, efficient extraction conditions for maximizing these compounds remain insufficiently established. This study aimed to optimize ultrasound‐assisted extraction (UAE) conditions for total phenolic content (TPC), DPPH radical scavenging activity, and reducing power from green mango peel using response surface methodology (RSM). A three‐factor, three‐level Box–Behnken design (BBD) was employed to investigate the effects of extraction time (10–30 min), temperature (30°C–60°C), and ethanol concentration (30%–70%). A total of 17 experimental runs were conducted, and second‐order polynomial models were developed for all responses. The adequacy of the models was confirmed by analysis of variance (ANOVA), which showed that all independent variables significantly influenced TPC, DPPH, and reducing power (*p* < 0.01). The models demonstrated strong statistical performance, with *R* ^2^ values of 0.9976, 0.9943, and 0.9989, and adjusted *R* ^2^ values of 0.9754, 0.9707, and 0.9827 for TPC, DPPH, and reducing power, respectively. The predicted *R* ^2^ values (0.8302, 0.7966, and 0.8794) further indicate good model reliability and acceptable predictive capability. RSM optimization identified the optimal extraction conditions as 36.25°C, 12 min, and 53.75% ethanol concentration. Under these conditions, the experimental values for TPC, DPPH radical scavenging activity, and reducing power were 28 ± 1.5 mg GAE/g DW, 58% ± 2%, and 46 ± 1.8 mg AAE/g DW, respectively, which closely matched the predicted values, confirming the validity of the developed models. Overall, the study demonstrates that UAE combined with RSM is an efficient, reliable, and green extraction strategy for recovering phenolic compounds from green mango peel. The optimized process supports the sustainable valorization of agro‐industrial waste and highlights the potential of mango peel as a functional ingredient source for antioxidant‐rich food and nutraceutical applications.

## Graphical abstract

Ultrasound‐assisted extraction (UAE) was applied to green mango peel to enhance the recovery of phenolic compounds and antioxidant activity. Response surface methodology was used to optimize extraction parameters, including temperature, time, and ethanol concentration. The optimized conditions significantly improved phenolic yield and antioxidant capacity, demonstrating the potential of mango peel as a sustainable source of natural bioactive compounds.

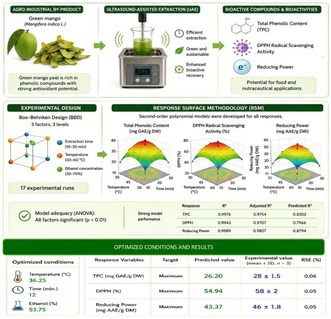

## 1 Introduction

Mango ( *Mangifera indica* L.) is a widely cultivated tropical fruit, valued for its nutritional, flavor, and culinary attributes. Global production exceeds 50 million metric tons annually, with India, Thailand, and Mexico as the leading producers (FAO 2023). Processing mangoes for juice and pulp generates substantial waste, particularly the peel, which accounts for 20%–40% of the fruit's weight (Tang et al. 2025). Green mango peel is rich in bioactive compounds such as phenolics, flavonoids, and vitamin C, exhibiting strong antioxidant, anti‐inflammatory, antimicrobial, and anticancer properties (Kučuk et al. 2024; Koirala et al. 2024; Tariq et al. 2023). These compounds, including gallic acid, catechin, and chlorogenic acid, contribute to free radical scavenging and metal chelation, making mango peel a valuable source of natural antioxidants for food, nutraceutical, and pharmaceutical applications (García‐Mahecha et al. 2023; Soria‐Lara et al. 2020).

The valorization of mango peel aligns with green chemistry principles, offering a sustainable solution to mitigate agro‐industrial waste pollution, including landfill accumulation and methane emissions from organic degradation (Hernandez‐Lozano et al. 2025). Green mango peel, a by‐product of mango processing, is a rich source of phenolic compounds and other bioactive substances with significant antioxidant potential. Recent studies have shown that mango peel contains among the highest total phenolic contents compared to other tropical fruit wastes (Suleria et al. 2020). Conventional extraction methods are often inefficient, consume large amounts of solvent, and can degrade heat‐sensitive bioactive compounds (Kučuk et al. 2024). Ultrasound‐assisted extraction (UAE) is an eco‐friendly and efficient alternative that enhances the recovery of phenolic compounds and antioxidants from green mango peel while reducing environmental impact (Kaur et al. 2022; Kumar et al. 2021). When key factors such as solvent composition, sonication time, and temperature are optimized, UAE can achieve significantly higher total phenolic content (TPC) and antioxidant activity compared to traditional methods (Christou et al. 2021; Tan Mei Chin et al. 2021). Response Surface Methodology (RSM) has been successfully applied to identify optimal extraction conditions. For example, conditions such as 54°C for 10 min using ethanol‐water mixtures have been reported to maximize the recovery of mangiferin, total phenols, and antioxidant capacity from mango peel (Ahmed et al. 2022; Ojeda et al. 2023; Morales et al. 2020).

Recent advances have also highlighted the benefits of using green solvents, particularly natural deep eutectic solvents, which enhance extraction yields and sustainability by reducing organic solvent usage and processing time (Lanjekar et al. 2022). In addition, ultrasound treatment induces microstructural disruption of the peel matrix through cavitation, improving the release of bound polyphenols and generating extracts with remarkable antioxidant and antimicrobial efficiency, making them valuable for food, nutraceutical, and cosmetic applications (Aznar‐Ramos et al. 2022; Sarker et al. 2024; Duan et al. 2026). Although the potential of the UAE for mango by‐products has been widely reported, there is still limited information concerning unripe (green) mango peel, which differs in phenolic composition and antioxidant profile from the ripe stage. Comprehensive optimization using RSM could provide deeper insight into the extraction behavior of these compounds and promote the sustainable utilization of mango processing waste as a source of natural antioxidants (Sarker et al. 2024; Ojeda et al. 2023; Morales et al. 2020).

Despite recent advances in the extraction of bioactive compounds from fruit processing by‐products, research specifically focused on green mango peel remains limited. Compared with ripe mango peel, green mango peel contains a distinct phenolic profile and often exhibits higher antioxidant potential due to the greater abundance of bioactive metabolites present during the unripe stage. Nevertheless, studies investigating the optimization of ultrasound‐assisted extraction (UAE) conditions for maximizing the recovery of these compounds from green mango peel are still scarce. In addition, the combined application of UAE and Response Surface Methodology (RSM) for the efficient extraction of phenolic compounds from this underutilized substrate has not been extensively reported. Therefore, the use of green mango peel in the present study represents a novel and sustainable approach for the valorization of agro‐industrial waste while promoting the development of cost‐effective natural antioxidant sources.

Accordingly, the present study aimed to optimize UAE parameters for the extraction of total phenolic compounds (TPC) and antioxidant activity, assessed through DPPH radical scavenging and reducing power assays, from green mango peel using RSM. The findings of this study may contribute to the development of efficient and environmentally sustainable extraction strategies for producing natural antioxidants with potential applications in the food, pharmaceutical, and cosmetic industries.

## 2 Materials and Methods

### 2.1 Collection and Preparation of Green Mango Peel Powder

Fresh green mangoes ( *Mangifera indica* L.) were procured from a local market in Pabna, Bangladesh. The fruits were selected at the unripe stage based on firm texture and green skin, as unripe mangoes are reported to contain higher levels of vitamin C, polyphenols, and antioxidant compounds (Lebaka et al. 2021). The fruits were thoroughly washed under running tap water to remove dust and surface contaminants. The peels were manually separated using a stainless‐steel peeler to avoid enzymatic browning and metal contamination, followed by rinsing with distilled water. The cleaned peels were subjected to sun drying under ambient conditions (35°C ± 5°C) for 2–3 days with occasional turning to ensure uniform drying. The final moisture content of the dried peel was 8.99 ± 0.9 g/100 g (dry weight basis), determined experimentally by oven drying at 105°C to constant weight. This confirms adequate dehydration for subsequent processing. Although sun drying is a simple and cost‐effective method, it may cause minor variability and partial degradation of heat‐ and light‐sensitive bioactive compounds. Therefore, it is considered a methodological limitation.

The dried peels were ground using a laboratory grinder (Panasonic MX‐AC400, Japan) and passed through a 0.5 mm mesh sieve to obtain a uniform particle size. This particle size was selected to ensure an optimal balance between increased surface area for efficient solvent penetration and acceptable material handling during extraction. Similar particle sizes have been widely used in plant‐based extraction studies to enhance mass transfer efficiency. For ultrasound‐assisted extraction (UAE), the powder was mixed with solvent at a solid‐to‐solvent ratio of 1:20 (w/v) (0.5 g sample in 10 mL solvent). This ratio was selected based on previously reported studies indicating that sufficient solvent volume is necessary. It ensures complete immersion of plant material and efficient extraction. The ultrasonic frequency used in the extraction process (40 kHz) has also been included to improve methodological reproducibility. The sieved mango peel powder (MPP) was finally stored in clean, air‐tight polyethylene bags at −18°C until further analysis. Although −80°C storage is often recommended for long‐term preservation of highly labile compounds, several studies have reported that −18°C is suitable for short‐term storage when samples are properly protected from light, oxygen, and moisture (Borges et al. 2023).

### 2.2 Chemicals and Reagents

All the chemicals and reagents employed were of analytical grade. Folin–Ciocalteu reagent, gallic acid, 1,1‐diphenyl‐2‐picrylhydrazyl (DPPH), potassium ferricyanide K_3_ [Fe(CN)_6_], and ferric chloride were purchased from Sigma‐Aldrich GmbH, Germany. Methanol, sodium carbonate, trichloroacetic acid (TCA), and phosphate buffer components were supplied by Merck, Germany. Distilled water was employed in all the experiments.

### 2.3 Response Surface Methodology (RSM)

To optimize the ultrasonic‐assisted extraction (UAE) conditions, a Box–Behnken Design (BBD) was employed within the framework of Response Surface Methodology (RSM) using Design‐Expert software (Version 13, Stat‐Ease Inc., Minneapolis, MN, USA). Based on preliminary trials and previous studies (Chemat et al. 2017), three independent variables were selected: ultrasonic time (A, 10–30 min), ultrasonic temperature (B, 30°C–60°C), and ethanol concentration (C, 30%–70% v/v). The experimental design consisted of 17 runs, including 12 factorial points and 5 center points, to ensure adequate model precision and minimize experimental error (Montgomery 2017). The responses measured were total phenolic content (TPC, expressed as mg gallic acid equivalents per g dry weight) and antioxidant activity, which included DPPH radical scavenging activity (expressed as % inhibition) and ferric reducing power (expressed as mg ascorbic acid equivalents per g dry weight).

The experimental data were fitted to a second‐order polynomial model:
(1)

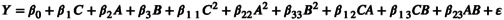

where: *Y* is the predicted response variable (e.g., total phenolic compounds, DPPH, reducing power). *β*_0_ is the intercept (constant term). *β*₁, *β*_2_, *β*_3_ are the linear regression coefficients corresponding to the independent variables *C*, *A*, and *B*. *β*₁₁, *β*_22_, *β*_33_ are the quadratic regression coefficients for the squared terms of the independent variables (*C* ^2^, *A* ^2^, *B* ^2^). *β*₁_2_, *β*₁_3_, and *β*_23_ are the interaction coefficients representing the effects of interactions between the independent variables (*CA*, *CB*, *AB*). *A* represents extraction time (minutes), *B* represents extraction temperature (°C). *C* represents ethanol concentration (%), *ε* (epsilon) is the random error term accounting for unexplained variation in the response.

Response surface methodology (RSM) was used to analyze the experimental data. Analysis of variance (ANOVA) was used to test the fitness of the model and the significance of the terms. It is very helpful to have a clear view of the relationship of the variables and their effect on the response, and 3D response surface plots were generated.

### 2.4 Optimization of Extraction Parameters Through Preliminary Trials

Preliminary trials were conducted to delineate the working ranges of the independent variables before formal optimization. In these trials, ethanol concentrations below 40% and above 80% yielded lower total phenolic recoveries, likely because extreme solvent polarities impair the solubility and mass transfer of phenolic compounds (dos Santos et al. 2020). For each candidate extraction temperature, five replicate samples were processed using a fixed extraction time of 30 min. It was further observed that extending extraction durations beyond 30 min or increasing temperatures above 60°C accelerated degradation of thermolabile compounds, especially vitamin C (ascorbic acid), consistent with known kinetics of ascorbic acid degradation at elevated temperatures (Akyildiz et al. 2021).

### 2.5 Ultrasound‐Assisted Extraction (UAE) Procedure

Ultrasound‐assisted extraction (UAE) of bioactive compounds from green mango peel powder was performed according to the experimental conditions defined by the Box–Behnken Design (BBD) (Table 1). For each extraction run, 0.5 g of mango peel powder was accurately weighed and mixed with 10 mL of ethanol solution of varying concentrations (30%, 50%, or 70% v/v) in a 100 mL glass beaker. The beaker was then placed in an ultrasonic bath, ensuring that the liquid level in the bath was maintained to completely immerse the sample. The ultrasonic bath was operated at a constant power of 100 W, with temperature and time settings adjusted according to the design matrix. During extraction, the ultrasonic waves generated cavitation effects that enhanced solvent penetration and mass transfer between the solvent and plant matrix. After completion of the extraction, the mixture was filtered through Whatman No. 1 filter paper to remove insoluble residues. The filtrate was subsequently centrifuged at 4000 rpm for 10 min to eliminate any remaining suspended particles. The clear supernatant was carefully collected, transferred into airtight amber vials to prevent light‐induced degradation, and stored at −18°C until further analysis.

**TABLE 1:** Experimental design matrix of Box–Behnken design (BBD) for ultrasound‐assisted extraction (UAE) optimization.

Independent variables	Symbol	Coded level		
		−1	0	1
Ultrasonic time (min)	A	10	20	30
Ultrasonic temperature (°C)	B	30	45	60
Ethanol concentration (%)	C	30	50	70

### 2.6 Determination of Proximate Composition

The proximate composition of the sample was determined according to specific AOAC Official Methods (2016). Moisture content was analyzed using AOAC Official Method 925.10 by drying 5 g of the sample in a hot air oven (Model ED 56, Tuttlingen, Germany) at 105°C until constant weight was achieved, and results were expressed as percentage weight loss. Ash content was determined using AOAC Official Method 923.03 by incinerating the dried sample in a muffle furnace at 550°C until a constant weight of ash was obtained, and the ash content was expressed as a percentage of the original sample weight. Crude fat was determined using AOAC Official Method 920.39 by Soxhlet extraction of 3 g of sample using petroleum ether (boiling range 40°C–60°C, analytical grade) for 6 h at 80°C. After extraction, the solvent was evaporated, and the residue was dried and weighed, and the fat content was expressed as a percentage of the sample weight. Crude protein content was determined using the Kjeldahl method (AOAC Official Method 978.04). Briefly, 1 g of sample was digested with concentrated H_2_SO_4_ in the presence of a catalyst mixture consisting of CuSO_4_ (0.2 g) and K_2_SO_4_ (1 g), along with 20 mL of distilled water, on a digestion block until white fumes appeared and digestion continued for 60–90 min until a clear solution was obtained with no residual charred material. After cooling, the digest was transferred to a distillation unit, and 50 mL of 32% NaOH was added to liberate ammonia, which was steam distilled for about 5 min into a receiving flask containing 4% boric acid solution. The distillate was titrated against 0.1 N H_2_SO_4_ using an appropriate indicator until the endpoint was reached, and a reagent blank was included for correction. Total nitrogen was calculated using the standard formula: % Nitrogen = [(V − V_0_) × N × 14 × 100]/W, where V is the sample titration volume, V_0_ is the blank, N is the normality of acid, and W is the sample weight. Crude protein content was then calculated as % Protein = % Nitrogen × 6.25.

### 2.7 Total Phenolic Content (TPC)

The determination of the total phenolic content (TPC) of each sample was carried out according to the method of Tahosin et al. (2025). 0.5 mL of the extract was combined with 0.5 mL of Folin–Ciocalteu's, and 7.5% Na_2_CO_3_ was added to the mixture for neutralization. After adding 8 mL of distilled water, the liquid was vortexed for 20 s before being left at room temperature for 35 min. After that, the sample was centrifuged for 10 min at 1968*g*, and the absorbance was measured at 725 nm with a spectrophotometer (UV/VIS, UV‐1800). The total phenolics were calculated by using an equation obtained from the gallic acid calibration curve (0–210 μg/g) and expressed as μg/g DW. All measurements were performed in triplicate, and results were expressed as mean ± standard deviation.

### 2.8 DPPH Antioxidant Activity

The free radical scavenging capacity was determined using the DPPH assay according to Halim et al. (2025) with some modifications. A solution of DPPH was freshly prepared by dissolving 6 mg DPPH in 50 mL of methanol (about 0.3 mM). The extract (1 mL) and DPPH solution (1.9 mL) were mixed in a test tube. The contents were then mixed thoroughly and kept in the dark for 30 min at room temperature. The absorbance was read at 517 nm using a spectrophotometer (UV/VIS, UV‐1800). The DPPH scavenging activity was measured using an ascorbic acid standard (0–260 μg/g) and expressed as micromoles equivalents per gram of DW of the sample. All measurements were performed in triplicate, and results were expressed as mean ± standard deviation.

### 2.9 Reducing Power Assay

The reducing power was carried out according to Halim et al. (2024) with slight modifications. The extracted sample (0.5 mL) was mixed with phosphate buffer (2.5 mL, 0.2 M, pH 6.6) and potassium ferricyanide (2.5 mL, 1% w/w) in a test tube, followed by incubating in a water bath at 50°C for 20 min. After that, trichloroacetic acid (2.5 mL, 10% w/v) was added to the tube and centrifuged (3424 g for 10 min). The supernatant (2.5 mL) was diluted with distilled water (2.5 mL), and freshly prepared ferric chloride (0.5 mL; 1%, w/w) was added. The mixture was mixed thoroughly, and its absorbance was read at 700 nm using a spectrophotometer (UV/VIS, UV‐1800, Japan). The FRAP assay was measured using an ascorbic acid standard (0–1000 μg/g), and the result was expressed as micromole Trolox equivalents (TE) per gram of DW of the sample. All measurements were performed in triplicate, and results were expressed as mean ± standard deviation.

### 2.10 Statistical Analysis

Statistical significance of the model and independent variables (*p* < 0.05) was determined with the aid of Design‐Expert software through analysis of variance (ANOVA). Model fit was assessed using the coefficient of determination (*R* ^2^) as well as adjusted *R* ^2^. Graphical analysis such as response surface and contour plots was plotted to depict the effects of independent variables on responses. Means of UAE and conventional maceration were compared with each other using Tukey's test (Montgomery 2017).

## 3 Results and Discussion

### 3.1 Physicochemical Properties of Mango Peel

The proximate composition of mango peel was determined on a dry weight (DW) basis, and the results are presented in Table 2. The analysis revealed that green mango peel contains appreciable amounts of moisture, ash, fat, and protein, highlighting its potential as a functional food ingredient and a source of bioactive compounds. The moisture content was 8.99 ± 0.9 g/100 g, indicating that the sample was relatively dry. This low moisture level is beneficial for enhancing shelf stability and reducing the risk of microbial growth during storage. Comparable values have been reported in the literature; for instance, Kučuk et al. (2024) reported a moisture content of 11.06% in completely air‐dried mango peels stored at room temperature in airtight containers, while Ibrahim et al. reported a value of 10.13%. The slightly lower moisture content observed in the present study may be attributed to differences in drying techniques, temperature, duration, and post‐drying handling conditions, as well as variations in mango variety and maturity stage. Similar findings have been reported in previous studies, further supporting the suitability of mango peel as a low‐moisture by‐product for powder formulation and extract concentration (Gichau et al. 2020; Vu et al. 2023). The relatively higher standard deviation (±0.9) may reflect minor inconsistencies in drying efficiency or sample heterogeneity; however, all analyses were conducted in triplicate under controlled laboratory conditions to ensure reliability of the results.

**TABLE 2:** Proximate composition of green mango peel on a dry weight basis.

Parameters	Value (g/100 g DW)
	Mean ± SD, *n* = 3
Moisture content	8.99 ± 0.9
Ash	3.09 ± 0.3
Fat	1.94 ± 0.1
Protein	3.76 ± 0.5

The ash content was 3.09 ± 0.3 g/100 g (DW), representing the total mineral fraction of the green mango peel. This moderate mineral content indicates the presence of essential inorganic nutrients such as calcium, potassium, and magnesium, which play important roles in physiological functions and antioxidant defense mechanisms (Razzaque and Wimalawansa 2025). The obtained value is consistent with recent literature reports on mango peel composition. For example, a recent study reported ash contents ranging from 1.85% to 2.93% in mango peel across different cultivars (Bamogo et al. 2025). Similarly, Baddi et al. (2015) reported an ash content of approximately 2.9% in mango peel powder, which closely aligns with the present findings. Comparable values have also been documented in other fruit by‐products, typically ranging from 2% to 5%, depending on cultivar, maturity stage, and processing conditions. Minor variations among studies may be attributed to differences in drying methods, environmental growing conditions, and post‐harvest handling. Overall, these comparisons confirm that mango peel is a mineral‐rich agro‐industrial by‐product with strong potential for functional food and bioactive applications.

The fat content was relatively low (1.94 ± 0.1 g/100 g, DW), which is typical for fruit by‐products. Despite the low lipid level, mango peel may still contain lipid‐soluble bioactive compounds such as carotenoids and certain phenolics, contributing to its antioxidant properties (Tariq et al. 2023). The obtained value is consistent with recent studies reporting similarly low fat levels in mango peel. For example, recent proximate analyses have shown fat contents ranging from approximately 1.5%–2.5% across different mango cultivars and processing conditions (Wongkaew et al. 2021). In addition, recent valorization studies have reported fat levels in mango peel between 1.6% and 3.7%, depending on variety, maturity stage, and processing conditions, further confirming that mango peel is a naturally low‐fat matrix (Ibrahim et al. 2025). Comparable findings have also been reported in other mango by‐product studies, where fat content is typically below 3%, supporting its suitability as a low‐fat agro‐industrial residue for functional food applications (Kamal et al. 2026). These similarities across studies confirm the reliability of the present results and support the potential use of mango peel in low‐fat functional food formulations.

The protein content was determined to be 3.76 ± 0.5 g/100 g (DW), indicating a moderate level of nitrogenous compounds in mango peel. This value suggests that mango peel contains plant‐derived proteins that may contribute to its functional and nutritional properties (Elsayed et al. 2022). The obtained value is consistent with recent literature reports on mango peel composition. A recent study reported protein content of approximately 2.05% (DW) in mango peel powder, confirming the presence of moderate nitrogenous compounds in this by‐product (Ganguly et al. 2025). Similarly, other proximate analyses have reported protein levels ranging from around 3% to 6.5%, depending on cultivar, maturity stage, and drying method (Jakha et al. 2024). These variations are mainly attributed to differences in mango variety, environmental growing conditions, and post‐harvest processing techniques. Overall, the present result falls within the commonly reported range for mango peel protein content, supporting its classification as a moderate but nutritionally relevant plant‐based protein source.

### 3.2 Fitting the Model and Analysis of Variance

The ultrasound‐assisted extraction (UAE) conditions affecting the phenolic content and antioxidant activity in green mango peel were optimized using the Box–Behnken design (BBD). Three variables (A: ultrasound time, min; B: temperature, °C; C: ethanol concentration, %) were studied in relation to total phenolic content (TPC), and their effects on the responses (TPC, DPPH radical scavenging activity, and reducing power) were examined. The experimental results (Table 3) were analyzed using a second‐order polynomial equation to correlate the independent and response variables. The developed regression equations are (2), (3), (4) as follows:

(2) $$\begin{array}{cc} Y_{\mathrm{TPC}\;\left(\mathrm{mg}\;\mathrm{GAE}/g\;\mathrm{DM}\right)}= & \;49.6+15.375\times A+5.2875\times B-1.7125\times C-0.2\\ & \;\times \mathrm{AB}-6.7\times \mathrm{AC}-9.525\times \mathrm{BC}-8.5125\\ & \;\times A^{2-}\;12.2875\times B^{2-}\;16.3875\times C^{2} \end{array}$$

(3) $$\begin{array}{cc} Y_{\text{DPPH}\;\left(\%\right)}= & \;86.5+15.675\times A+10.6125\times B-3.5125\times C+2.25\\ & \;\times \mathrm{AB}-14.25\times \mathrm{AC}-1.175\times \mathrm{BC}-16.4125\\ & \;\times A^{2}-14.5375\times B^{2}-22.6375\times C^{2} \end{array}$$

(4) $$\begin{array}{cc} Y_{\text{Reducing Power}\;\left(\mathrm{mg}\;\mathrm{AAE}/g\;\mathrm{DM}\right)}= & \;86.1+26.325\times A+10.425\times B-7.2\times C+1.5\\ & \;\times \mathrm{AB}-11.4\times \mathrm{AC}-10.2\times \mathrm{BC}-15.3\\ & \;\times A^{2}-22.5\times B^{2}-36.3\times C^{2} \end{array}$$

**TABLE 3:** Box–Behnken design (BBD) matrix for three variables and corresponding experimental response values.

Run	Independent variables			Response variables		
				Antioxidant activity		
	A	B	C	TPC (mg GAE/g DM)	DPPH (%)	Reducing power (mg AAE/g DM)
1	30	45	30	45.2	78.2	76.2
2	20	45	50	49.8	86.5	86.1
3	20	60	30	38.5	62.1	54.6
4	30	60	50	51.5	89.3	90.3
5	30	45	70	30.1	42.8	37.5
6	10	60	50	16.3	48.2	26.7
7	20	45	50	50.1	87.1	86.7
8	30	30	50	41.7	58.4	66.9
9	20	45	50	48.9	85.8	85.5
10	20	30	70	22.4	38.9	20.4
11	10	45	70	17.6	45.2	15.6
12	20	60	70	14.3	52.6	21.3
13	20	30	30	8.5	43.7	12.9
14	20	45	50	49.5	86.2	85.8
15	10	45	30	5.9	23.6	8.7
16	20	45	50	49.7	86.9	86.4
17	10	30	50	5.7	26.3	9.3

The model terms' coefficients express the size and direction of the effect size of the model terms on the response. Positive coefficients indicate a synergistic effect, and negative coefficients indicate an antagonistic effect. For example, in the TPC model, the effect of *A* (ultrasound time) and *B* (temperature) was significant in a positive linear relationship, which could mean that more ultrasound time and temperature improve phenolic extraction. While *C* (ethanol) presented a negative linear effect, indicating that the higher the ethanol concentration, the lower the TPC. The same tendency was observed for DPPH and reducing power since *A* and *B* have a positive effect on the antioxidant activity and C has a negative influence. The quadratic terms (*A* ^2^, *B* ^2^, *C* ^2^) of all the models were significant, indicating a non‐linear behavior of the main effects terms in the responses. The interaction terms (e.g., *AC*, *BC*) were also remarkable, especially in power reduction, where *AC* and *BC* displayed significant negative interactions.

UAE considerably reduces extraction time, energy consumption, and solvent usage while preserving heat‐sensitive compounds compared with conventional extraction techniques such as maceration, infusion, and decoction (Picot‐Allain et al. 2021; Pillai et al. 2024). Experimentally (Table 3), the highest total phenolic content (TPC) (51.5 mg GAE/g DW), DPPH radical scavenging activity (89.3%), and reducing power (90.3 mg AAE/g DW) were obtained in Run 4 (A: 30 min, B: 60°C, C: 50% ethanol). This represents the best observed experimental response within the design space. In contrast, the lowest values for all responses were observed in Run 15 (A: 10 min, B: 45°C, C: 30% ethanol), indicating the necessity of optimizing extraction conditions.

These findings are consistent with previous studies on ultrasound‐assisted extraction of phenolic compounds, which reported that moderate‐to‐high temperatures (50°C–70°C) and intermediate ethanol concentrations (40%–60%) enhance extraction efficiency due to improved solvent penetration and cell wall disruption (Taweekayujan et al. 2023; Šavikin et al. 2021). However, excessively high ethanol concentrations (> 70%) may reduce extraction efficiency by lowering solvent polarity and thereby limiting phenolic solubility (Dent et al. 2021).

The high *R* ^2^ value confirms that the developed model adequately predicts the responses within the experimental range. The RSM analysis identified the optimal region within the design space corresponding closely to the experimental optimum (Run 4 conditions), indicating strong agreement between predicted trends and observed data. This consistency confirms the reliability of the model for describing the extraction behavior of phenolic compounds from green mango peel. Similarly, recent studies employing response surface methodology and artificial neural network approaches for microwave‐assisted extraction of mango peel bioactives have reported strong agreement between experimental and predicted values, further supporting the robustness of model‐based optimization (Ramírez‐Brewer et al. 2024).

### 3.3 Model Adequacy, ANOVA, and RSM Optimization

The results of analysis of variance (ANOVA) (Table 4) demonstrated that all three quadratic models were highly significant, as indicated by large *F*‐values (TPC: 71.54; DPPH: 59.86; reducing power: 101.76) and very low *p*‐values (*p* < 0.0001). These results confirm that ultrasound‐assisted extraction (UAE) time (A), temperature (B), and ethanol concentration (C) significantly influence total phenolic content (TPC), DPPH radical scavenging activity, and reducing power, validating the suitability of Response Surface Methodology (RSM) for modeling and optimization within the experimental domain. The linear terms of ultrasound time (A) and temperature (B) were highly significant (*p* < 0.01) across all responses, indicating their strong positive effect on extraction efficiency, likely due to enhanced cavitation, improved mass transfer, and effective cell wall disruption. In contrast, ethanol concentration (C) showed a variable effect, being non‐significant for TPC (*p* = 0.1249) but significant for antioxidant responses (DPPH: *p* = 0.0405; reducing power: *p* = 0.0022), suggesting that solvent polarity plays a more important role in modulating antioxidant activity than total phenolic yield alone. Significant interaction effects (*AC* and *BC*) indicated that ethanol concentration interacts with both ultrasound time and temperature, whereas the non‐significant AB interaction suggests that time and temperature act largely independently within the studied range. The significance of quadratic terms (*A* ^2^, *B* ^2^, and *C* ^2^; *p* < 0.0001) confirmed the non‐linear nature of the system and the existence of true optimal regions, beyond which further increases in process variables may reduce extraction efficiency due to degradation or saturation effects.

**TABLE 4:** Analysis of variance (ANOVA) of the quadratic models for total phenolic content and antioxidant activity.

Source	TPC	DPPH	Reducing power
*A*	< 0.0001*	< 0.0001*	< 0.0001*
*B*	0.0010*	0.0001*	0.0003*
*C*	0.1249	0.0405*	0.0022*
*AB*	0.8896	0.2933	0.5122
*AC*	0.0019*	0.0002*	0.0012*
*BC*	0.0002*	0.5716	0.0022*
*A* ^2^	0.0004*	< 0.0001*	0.0002*
*B* ^2^	< 0.0001*	0.0001*	< 0.0001*
*C* ^2^	< 0.0001*	< 0.0001*	< 0.0001*
*R* ^2^	0.9892	0.9872	0.9924
Adjusted *R* ^2^	0.9754	0.9707	0.9827
Predicted *R* ^2^	0.8302	0.7966	0.8794
*F*‐value	71.54*	59.86*	101.76*
Prob > *F*	< 0.0001*	< 0.0001*	< 0.0001*
C.V. (%)	8.66	6.46	8.48
Lack of fit	0.0004*	0.0002*	< 0.0001*

* Significant at *p* < 0.05. *A*, *B*, *C*: Independent variables (coded factors). *R* ^2^: Coefficient of determination; Adjusted *R* ^2^: Adjusted for model complexity; Predicted *R* ^2^: Predictive capability. C.V., coefficient of variation (measure of experimental precision). Lack of fit: Significant values indicate poor model fit to experimental data.

The models demonstrated strong goodness of fit, with coefficients of determination (*R* ^2^ > 0.98) indicating that more than 98% of the variability in responses was explained. The close agreement between adjusted and predicted *R* ^2^ values confirmed minimal overfitting, while low coefficients of variation indicated good reproducibility. However, despite these strong statistical indicators, the significant lack‐of‐fit (*p* < 0.001) suggests that the quadratic models did not fully capture all sources of variability. This limitation may arise from inherent experimental variability, unaccounted higher‐order interactions, or complex matrix effects typical of plant‐based systems. Therefore, although the models are reliable within the studied range, caution is required when interpreting predictions beyond the experimental domain.

A clear distinction must be made between the highest experimental responses and the optimized conditions obtained through the desirability function approach. Although the highest experimental responses were obtained in Run 4 (30 min, 60°C, 50% ethanol), these values represent single‐response maxima within the experimental design space. In contrast, the optimized conditions were selected based on simultaneous optimization of all responses (TPC, DPPH, and reducing power) using a desirability function approach. This method identifies a statistically balanced solution rather than maximizing individual responses, and therefore provides a more practically applicable operating condition. From a statistical perspective, the desirability‐based optimum ensures simultaneous improvement across all responses and reduces bias toward any single parameter. From a practical perspective, it offers improved process robustness, reproducibility, and potential scalability, even if individual response values are slightly lower than the absolute experimental maximum.

Model validation confirmed that Run 4 produced the highest experimental responses (TPC: 51.5 mg GAE/g DW; DPPH: 89.3%; reducing power: 90.3 mg AAE/g DW), which were in close agreement with the overall predicted trends within the design space. This indicates that moderate ethanol concentration combined with higher temperature and sufficient ultrasound exposure enhances phenolic extraction and antioxidant activity. Furthermore, the present study demonstrates substantially higher extraction efficiency compared to conventional techniques. For example, Gunathilake et al. (2019) reported a maximum TPC yield of 4.71 mg/g using maceration extraction, which is considerably lower than the values obtained in this study. This improvement is attributed to ultrasound‐induced cavitation, where bubble formation and collapse generate microjets and shear forces that disrupt plant cell structures, thereby enhancing solvent penetration and release of bound phenolic compounds. This mechanism has been widely reported to improve bioactive recovery compared with conventional extraction methods (Rodríguez De Luna et al. 2020; Hao et al. 2023; Milićević et al. 2021).

### 3.4 Effect of Independent Variables on the TPC

**FIGURE 1:**
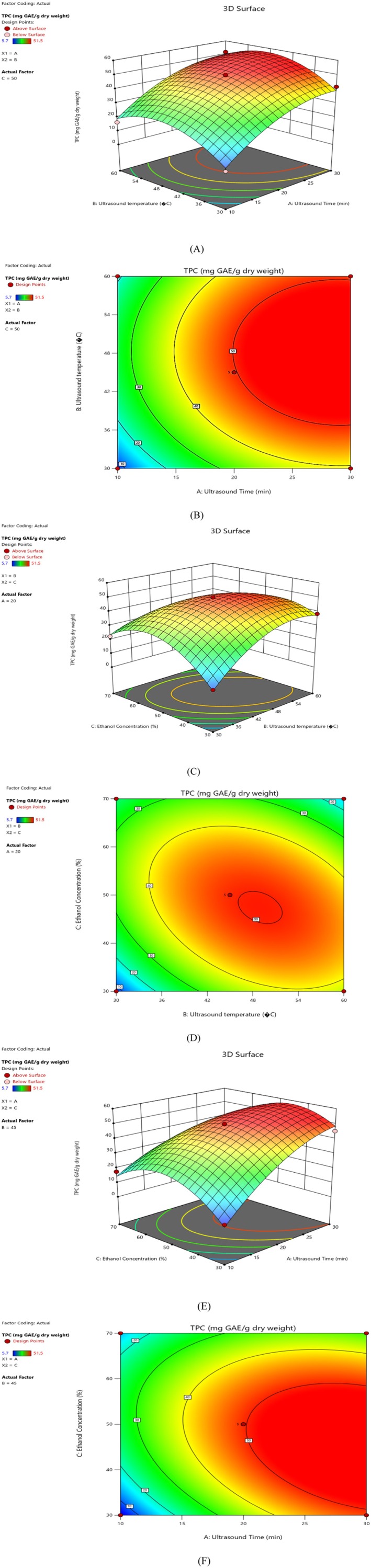
Response surface and contour plots illustrating interaction effects of extraction time and temperature (A, B), temperature and ethanol concentration (C, D), and time and ethanol concentration (E, F) on total phenolic content (TPC) of green mango peel.

Response Surface Methodology demonstrated that extraction parameters significantly influenced the total phenolic content (TPC) of mango peel. The three‐dimensional response surface plots (Figure 1A–F) illustrate the complex interactions among ultrasound time, temperature, and ethanol concentration on extraction efficiency. The elliptical contour patterns observed in Figure 1A,B indicate a significant interaction between ultrasound time and temperature, with maximum TPC achieved at intermediate conditions (approximately 30 min and 60°C). From a mechanistic perspective, increasing temperature enhances solvent diffusivity, reduces viscosity, and accelerates mass transfer, thereby improving the release of phenolic compounds. Simultaneously, ultrasound induces cavitation, generating microjets and shear forces that disrupt plant cell walls and facilitate the liberation of bound phenolics (Lama‐Muñoz and Contreras 2022). However, excessive temperature or prolonged sonication may promote oxidation, structural degradation, or polymerization of heat‐sensitive phenolic compounds, ultimately reducing extraction efficiency (Dzah et al. 2020). This interplay between enhanced mass transfer and potential degradation explains the existence of an optimal extraction region and the decline in TPC beyond critical processing conditions. The statistical significance of the quadratic terms (*p* < 0.0001) in Equation 2 further confirms the non‐linear behavior of the system, indicating that optimal conditions exist within a defined range and that deviations from these conditions adversely affect phenolic recovery. These findings are consistent with previously reported studies on ultrasound‐assisted extraction of plant bioactives (Wang et al. 2024).

The second piece interaction between temperature and ethanol concentration (Figure 1C,D) behavior was especially interesting because there the highest phenolic transfer values were reached at moderate ethanol concentration conditions (40%–60%) and the temperature conditions of 60°C, which is in accordance with Azmir et al. (2013), who reasoned that the combination allows for an optimal exposure of solvents about their polarity for phenolic solubility and compounds stability. The negative BC interaction term (−9.525) would imply that high levels of ethanol are offset as a detriment at high temperatures, probably due to faster degradation of heat‐labile phenolics in non‐polar environments. The time‐ethanol interaction Figure 1E,F shows that longer extraction times (> 35 min) in combination with high ethanol contents (> 70%) led to the poorest TPC yields, which is in line with the argumentation of Kumar et al. (2021) that prolonged contact with nonpolar solvents might result in phenolic breakdown and extraction failure.

Its *R* ^2^ value is very high (0.9892), supported by a significant *F*‐value (71.54) (*p* < 0.0001), indicating a good predictability of the model in predicting TPC yield under different extraction conditions. Its highly significant lack‐of‐fit (*p* = 0.0004) indicates that there might be additional small causes influencing phenolic extraction not explained by the current model. Taken together, these results indicate TPC from the mango peel is best extracted by compromising all three parameters, and the maximum yields would be obtained at about 30 min ultrasonic time, 60°C, and 50% ethanol. These results are relevant to industrial extraction processes and confirm that for the recovery of phenolic compounds from mango peel by‐product, mild instead of hard conditions are more favorable.

## 4 Response Surface Analysis of Antioxidant Activities

### 4.1 Effect of Extraction Parameters on DPPH Radical Scavenging Activity

**FIGURE 2:**
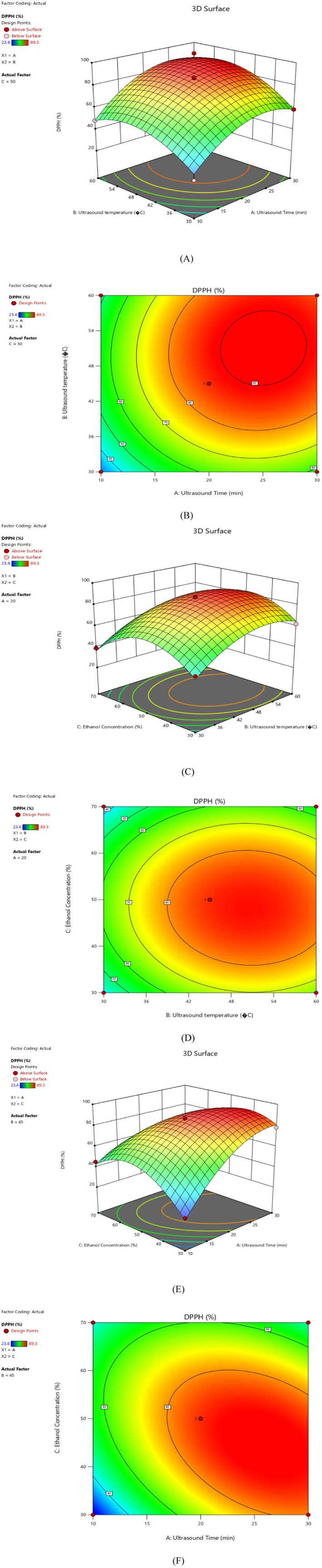
Response surface and contour plots illustrating interaction effects of extraction time and temperature (A, B), temperature and ethanol concentration (C, D), and time and ethanol concentration (E, F) on DPPH radical scavenging activity of green mango peel extract.

The optimization of ultrasound‐assisted extraction conditions for DPPH radical scavenging activity from mango peel revealed significant relationships between process variables and antioxidant potential (Figure 2A–F). The quadratic model demonstrated excellent predictive capability (*R* ^2^ = 0.9872), with all model terms highly statistically significant (*F*‐value = 59.86, *p* < 0.0001). These findings align with previous research by Ghasemzadeh et al. (2018), who reported similar model adequacy in optimizing phenolic compound extraction from mango peel using response surface methodology.

Ultrasound time emerged as the most influential parameter (*p* < 0.0001), with activity increasing up to 30 min of extraction. This observation supports the findings of Zhang et al. (2025) in their study of ultrasonic‐assisted extraction from *Panax notoginseng* Leaves, where they attributed such improvements to enhanced cavitation effects that promote more thorough cell wall disruption and release of bound antioxidants. However, the significant quadratic term (*A* ^2^, *p* < 0.0001) confirms that excessive sonication leads to degradation of antioxidant compounds, consistent with reports by Ahmed et al. (2022) on thermal and oxidative degradation of polyphenols in plant extracts.

The temperature effect (*p* = 0.0001) showed optimal activity at 60°C, beyond which thermal degradation became significant (*B* ^2^, *p* = 0.0001). This temperature threshold agrees with findings by Kumar et al. (2021) in their comprehensive study of ultrasound‐assisted extraction parameters for mango peel polyphenols, where they observed similar thermal degradation patterns. Ethanol concentration exhibited a complex relationship, with moderate concentrations (40%–60%) providing optimal results, as previously observed by Santos et al. (2019) in their systematic investigation of solvent effects on phenolic extraction efficiency and antioxidant preservation.

The significant AC interaction (*p* = 0.0002) revealed that high ethanol concentrations (> 70%) diminish the benefits of extended extraction times, likely due to reduced solvent polarity limiting antioxidant solubility. This finding corroborates the extensive work of Azmir et al. (2013) on solvent selection principles for bioactive compound extraction from plant materials. The model predicted maximum DPPH activity (89.3%) at 30 min, 60°C, and 50% ethanol concentration, representing conditions that optimally balance extraction efficiency with compound stability, as suggested by Yuan et al. (2016) in their fundamental studies on extraction parameter optimization for plant phenolics.

### 4.2 Effect of Extraction Parameters on Reducing Power

**FIGURE 3:**
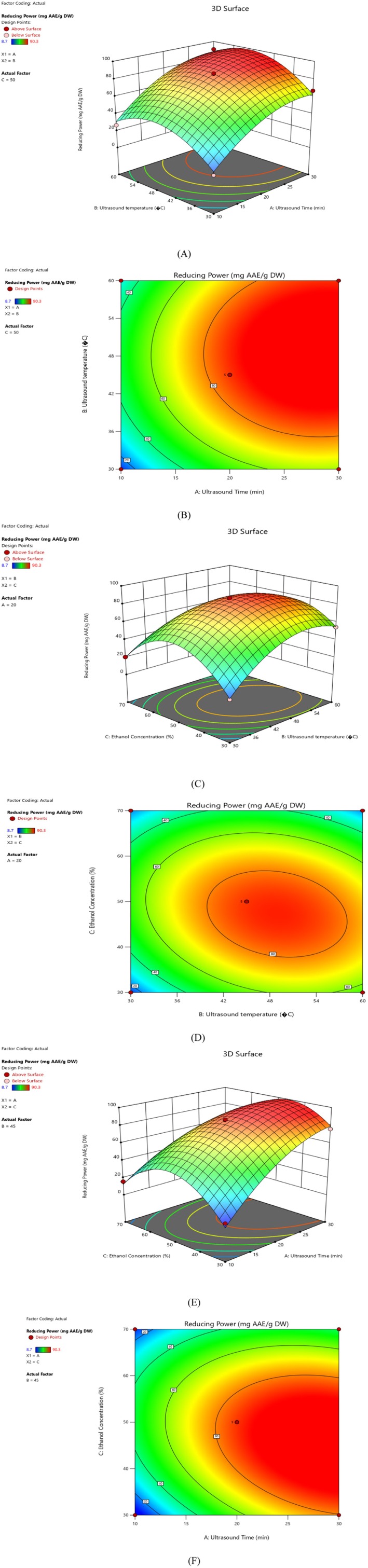
Response surface and contour plots illustrating interaction effects of extraction time and temperature (A, B), temperature and ethanol concentration (C, D), and time and ethanol concentration (E, F) on reducing power of green mango peel extract.

The study investigated the effect of extraction parameters: time (A), temperature (B), and ethanol concentration (C), on the reducing power of green mango peel, a key indicator of its antioxidant potential (Figure 3A–F). The response surface methodology (RSM) analysis revealed significant interactions among these variables, as evidenced by the high *R* ^2^ value of 0.9924 for reducing power, indicating the model explained over 99% of the variability in the data. This strong correlation is comparable to findings by Bezerra et al. (2008), who reported similar *R* ^2^ values (0.94–0.99) in their RSM optimization of phenolic compound extraction from plants. The adjusted *R* ^2^ (0.9827) and predicted *R* ^2^ (0.8794) further confirmed the model's robustness, with these metrics aligning with the criteria suggested by Myers et al. (2004) for adequate model precision in extraction optimization studies.

The ANOVA results demonstrated significant effects (*p* < 0.05) for all linear terms (*A*, *B*, *C*), interaction terms (AC, BC), and quadratic terms (*A* ^2^, *B* ^2^, *C* ^2^), except for the AB interaction (*p* = 0.5122). The exceptionally high *F*‐value of 101.76 (*p* < 0.0001) and significant lack of fit (*p* < 0.0001) indicated excellent model adequacy, consistent with the model validation standards proposed by Box and Draper (1987) for chemical extraction processes. The regression equation (Equation 1) revealed that time (A) and temperature (B) had positive linear coefficients (+26.325 and +10.425, respectively), suggesting their enhancing effects on reducing power. This aligns with the findings of Castañeda‐Valbuena et al. (2021), who observed similar time–temperature synergies in their study of antioxidant extraction from mango byproducts.

The negative linear coefficient for ethanol concentration (−7.2) and significant negative quadratic terms (*A* ^2^ = −15.3, *B* ^2^ = −22.5, *C* ^2^ = −36.3) revealed important non‐linear relationships. These results corroborate the findings of Lohvina et al. (2021), who demonstrated that ethanol concentrations beyond 70% become less effective for polar antioxidant extraction due to reduced solubility. The observed thermal enhancement threshold also supports Chemat et al. (2017) ultrasonication studies, which identified 60°C–70°C as optimal before thermal degradation occurs.

The three‐dimensional (3D) response surface plots provided valuable insights into the interactive effects among extraction parameters. The time–temperature interaction exhibited an initial rise followed by a plateau in reducing power, consistent with the thermal degradation patterns of phenolics reported in mango and citrus peel extracts (Wang et al. 2024). The temperature ethanol interaction revealed optimal extraction efficiency within the 50%–70% ethanol range, aligning with the findings of Patra et al. (2022), who observed that moderate ethanol concentrations (55%–65%) provide the best balance between solvent polarity and water activity for phenolic solubilization. Conversely, the time–ethanol interaction showed a decline in extraction efficiency at high ethanol levels (> 90%), corroborating the solvent polarity theory described by Xiang et al. (2024) and further supported by Osorio‐Tobón (2020), who noted that excessive ethanol reduces solvent polarity and limits phenolic diffusion from plant tissues. Moreover, comparable solvent‐related limitations were observed in Moringa and other fruit peel matrices, where UAE markedly enhanced phenolic recovery relative to conventional extraction methods (Wang et al. 2024).

These comprehensive findings not only validate the current optimization model but also extend the work of previous researchers by quantifying specific parameter interactions for mango peel extracts. The results suggest that industrial applications should target moderate ethanol concentrations (50%–70%) and controlled temperatures (likely 50°C–60°C based on the quadratic terms) for optimal antioxidant recovery, building upon the foundational work of Sayem et al. (2024) on mango byproduct valorization.

### 4.3 Optimization of Extraction Parameters and Model Validation

**FIGURE 4:**
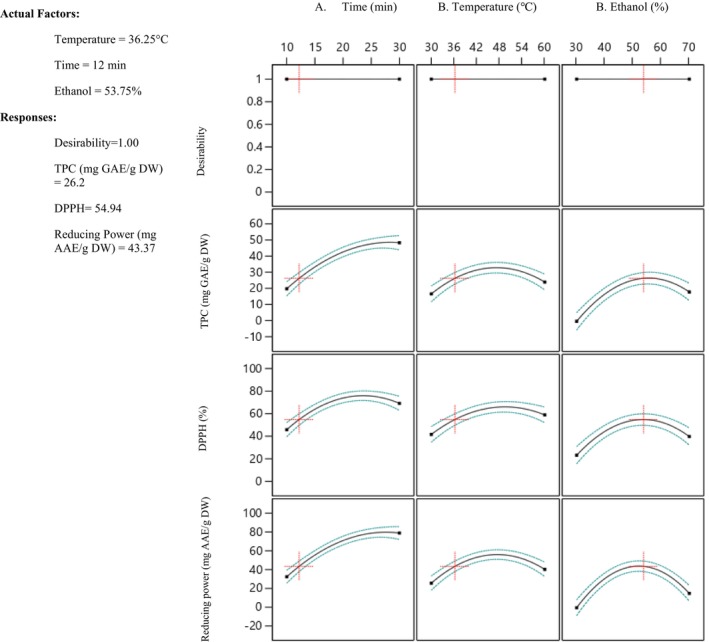
Response surface methodology (RSM)–based optimization of ultrasound‐assisted extraction conditions for mango peel bioactive compounds.

**FIGURE 5:**
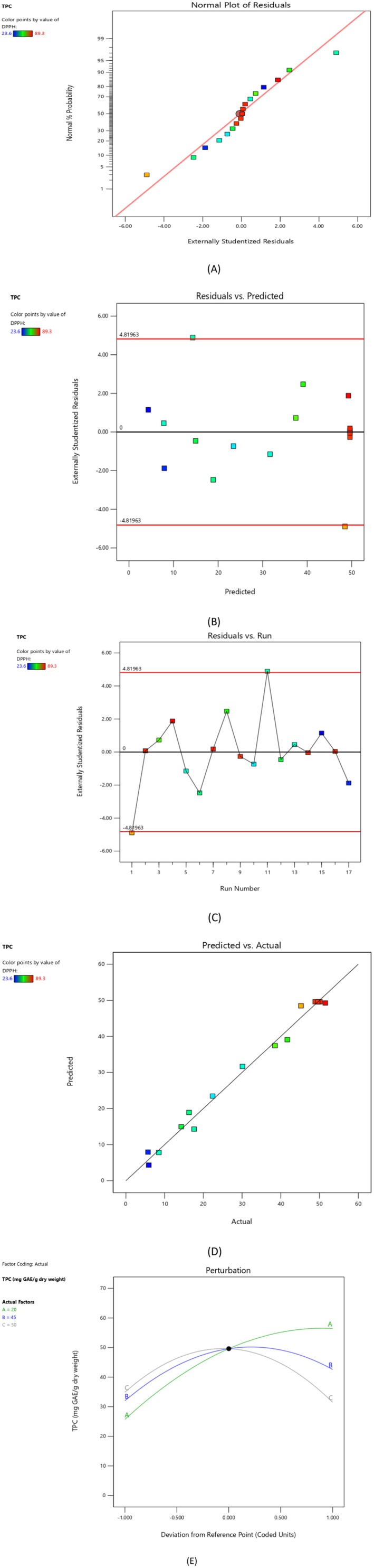
Diagnostic plots for validation of the RSM model for TPC: (A) Normal probability plot, (B) residuals versus predicted, (C) residuals versus run, (D) predicted versus actual, and (E) perturbation plot.

**FIGURE 6:**
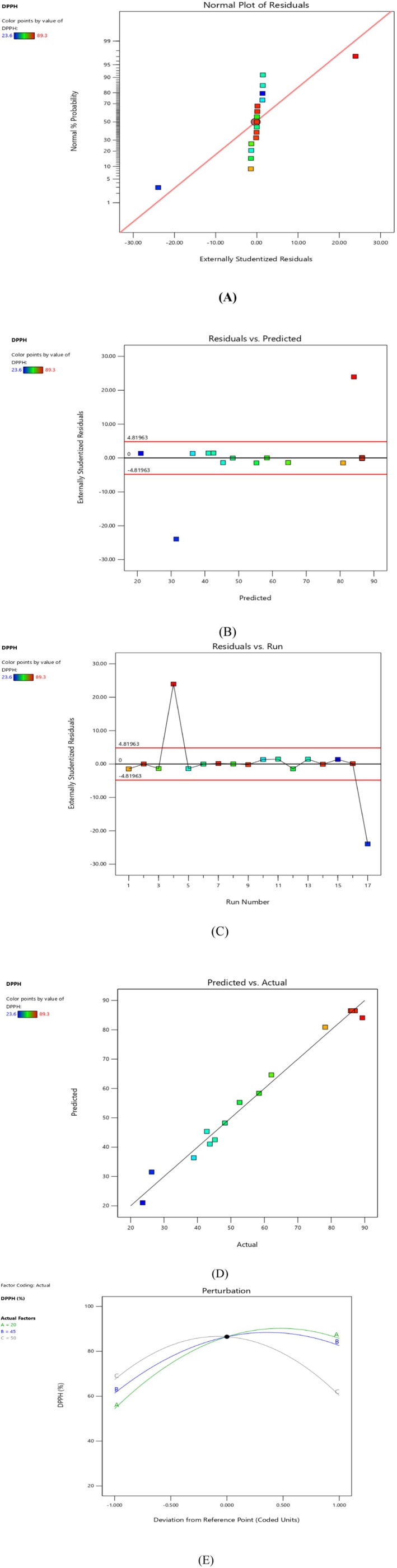
Diagnostic plots for validation of the RSM model for DPPH activity: (A) Normal probability plot, (B) residuals versus predicted, (C) residuals versus run, (D) predicted versus actual, and (E) perturbation plot.

**FIGURE 7:**
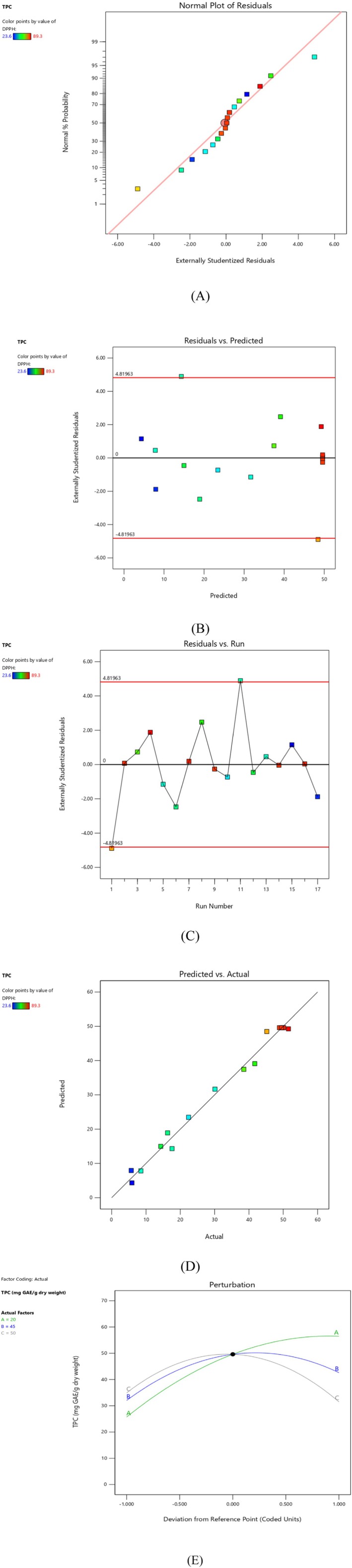
Diagnostic plots for validation of the RSM model for reducing power: (A) Normal probability plot, (B) residuals versus predicted, (C) residuals versus run, (D) predicted versus actual, and (E) perturbation plot.

One of the primary objectives of the present study was to determine the optimal process parameters for ultrasound‐assisted extraction (UAE) of green mango peel, specifically temperature, extraction time, and ethanol concentration, to maximize total phenolic content (TPC) and antioxidant activities. However, achieving maximum responses under identical conditions is challenging, as each response has a distinct optimal region. Therefore, a multi‐response optimization approach was required. In response surface methodology (RSM), two common optimization strategies are typically employed. The first involves the superimposition of contour plots and manual identification of optimal conditions; however, this approach is considered inefficient and difficult to automate (Granato et al. 2010). Consequently, the desirability function approach was applied using Design‐Expert software (version 12.0.3) to simultaneously optimize all responses. In this method, desirability values ranging from 0 (undesirable) to 1 (fully desirable) were assigned to each response, and the optimal conditions were determined by maximizing the overall desirability. The optimization process resulted in an overall desirability value of 1.00 (Figure 4), corresponding to optimal extraction conditions of 36.25°C, 12 min, and 53.75% ethanol concentration. Under these conditions, the predicted values were 26.20 mg GAE/g DW for TPC, 54.94% for DPPH radical scavenging activity, and 43.37 mg AAE/g DW for reducing power. To validate the model, experiments were conducted under the optimized conditions, and the responses were measured in triplicate. The experimental values obtained were 28 ± 1.5 mg GAE/g DW for TPC, 58% ± 2% for DPPH, and 46 ± 1.8 mg AAE/g DW for reducing power (Table 5). The close agreement between predicted and experimental values, with deviations of less than 7%, confirms the reliability and predictive capability of the developed models. The residual standard error (RSE) was used to evaluate the agreement between predicted and experimental results. The low RSE values (< 0.06%) indicate excellent model accuracy and robustness, consistent with previously reported validation criteria (Bezerra et al. 2008). The minor deviations observed can be attributed to inherent variability in plant‐based materials (Montgomery 2017). The adequacy of the models was further assessed using diagnostic plots. The normal probability plots of residuals indicated that the errors were normally distributed, while plots of residuals versus predicted values showed no significant trends, confirming the absence of systematic bias. Additionally, perturbation plots highlighted the relative influence of process variables on each response. The diagnostic plots for TPC, DPPH, and reducing power are presented in Figures 5A–E, 6A–E and 7A–E, respectively, demonstrating strong agreement between predicted and experimental values and confirming the adequacy of the developed quadratic models. Overall, the results demonstrate that the optimized UAE conditions effectively enhance the extraction of phenolic compounds and antioxidant activity from green mango peel. The strong correlation between predicted and experimental values, along with low RSE values, highlights the robustness of the model and supports its potential application for industrial‐scale extraction and valorization of agro‐industrial waste.

**TABLE 5:** Experimental values of TPC, DPPH, and reducing power under the optimized conditions.

Optimized conditions	Temperature ( 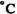 )	36.25			
	Time (min)	12			
	Ethanol (%)	53.75			
		Target	Predicted value	Experimental value	RSE (%)
Response variables	TPC (mg GAE/g DW)	Maximum	26.20	28 ± 1.5	0.06
	DPPH (%)	Maximum	54.94	58 ± 2	0.05
	Reducing power (mg AAE/g DM)	Maximum	43.37	46 ± 1.8	0.05

*Note:* Experimental values expressed as mean standard deviation of the mean (*n* ± 3).

Abbreviation: RSE, residual standard error.

## 5 Conclusion

The present work aimed to determine optimal conditions for maximizing TPC, DPPH radical scavenging activity, and reducing power. Response surface methodology (RSM) was effectively applied to evaluate the individual and interactive effects of extraction temperature, time, and ethanol concentration. The developed models demonstrated satisfactory predictive accuracy, and the optimal conditions were identified using a desirability function and subsequently validated experimentally. The optimization process focused on maximizing phenolic yield and antioxidant capacity, resulting in optimal conditions of 36.25°C, 12 min, and 53.75% ethanol. These conditions can be further adjusted depending on specific industrial or economic requirements to achieve desired outcomes. It is also important to note that additional ultrasound parameters, such as power, frequency, and duty cycle, may influence extraction efficiency and should be explored in future studies. Overall, this study provides a foundational approach for the efficient extraction and utilization of bioactive compounds from green mango peel, supporting its potential application in functional foods and nutraceuticals, and contributing to the sustainable valorization of agro‐industrial waste.

## 6 Limitations and Future Research Directions

Despite the strong statistical performance of the developed models, the significant lack‐of‐fit suggests that some variability was not fully captured, likely due to complex matrix effects or unmodeled higher‐order interactions. In addition, the absence of detailed phytochemical profiling (e.g., HPLC or LC–MS) limited the identification of specific bioactive compounds responsible for antioxidant activity. Future studies should focus on advanced compound characterization, comparison with other green extraction techniques, and evaluation of scale‐up feasibility, including techno‐economic and energy efficiency assessments, to support industrial applications.

## Author Contributions

**Mouluda Sohany:** investigation, writing – review and editing, supervision. **Md. Naimur Rahman:** writing – original draft, methodology, software, data curation, writing – review and editing. **Md. Sultan Mahomud:** conceptualization, investigation, writing – review and editing, validation, project administration, supervision. **Md. Arifur Rohman:** writing – original draft, methodology, formal analysis, software, data curation. **Md. Abdul Halim:** writing – original draft, writing – review and editing, software, data curation, formal analysis, resources.

## Funding

The authors have nothing to report.

## Ethics Statement

The authors have nothing to report.

## Conflicts of Interest

The authors declare no conflicts of interest.

## Data Availability

The datasets analyzed during the current study are available from the corresponding author upon reasonable request.
